# Sustained HBsAg clearance induced by pegylated interferon α-2b in HBeAg-negative patients with low baseline HBsAg

**DOI:** 10.3389/fcimb.2026.1803818

**Published:** 2026-05-20

**Authors:** Chaojing Wen, Taiyu He, Guanhua Zha, Aoyi Li, Zhiyi Wang, Zhi Zhou, Dachuan Cai, Dazhi Zhang, Mingli Peng, Xiaofeng Shi, Hong Ren

**Affiliations:** Department of Infectious Diseases, Key Laboratory of Molecular Biology for Infectious Diseases (Ministry of Education), Institute for Viral Hepatitis, the Second Affiliated Hospital, Chongqing Medical University, Chongqing, China

**Keywords:** chronic hepatitis B, functional cure, low HBsAg level, pegylated interferon α-2b, sustained HBsAg clearance

## Abstract

**Background:**

Patients with low HBsAg levels, HBeAg-negative status and undetectable HBV DNA are recognized as the advantaged population for pegylated interferon α-2b (Peg-IFNα-2b) therapy. However, there are limited reports on the sustainability of HBsAg loss in this population. This study aims to explore the patterns of sustained response following HBsAg clearance in low HBsAg level patients treated with Peg-IFNα-2b through long-term follow-up, providing guidance for clinical practice.

**Methods:**

This retrospective study analyzed patients with baseline HBsAg < 200 IU/mL, HBeAg negativity and undetectable baseline HBV DNA, who achieved HBsAg clearance and received either Peg-IFNα-2b monotherapy or nucleos(t)ide analog (NA) combination therapy. HBV biomarkers and clinical biochemical indicators were assessed during follow-up.

**Results:**

We evaluated sustained HBsAg loss and investigated the risk factors of HBsAg reversion. A total of 203 patients with baseline HBsAg<200IU/ml, HBeAg-negativity and undetectable HBV DNA were enrolled in this study. The median follow-up duration was 96 weeks (range:72 to 120 weeks). 44 (21.67%) patients were observed HBsAg reversion during follow-up, 91.36% (40/44) of which occurred within 96 weeks. Kaplan-Meier stratification analysis revealed that higher end-of-treatment (EOT) HBsAb levels and longer consolidation therapy duration were independently associated with higher sustained HBsAg clearance rates (P< 0.001). Specifically, patients with EOT HBsAb >1000 IU/mL achieved a 100% sustained clearance rate (no reversion observed). Cox regression analysis identified EOT HBsAb levels ≥100 IU/mL (HR = 0.238, P = 0.003)) and consolidation therapy ≥12 weeks (HR = 0.492, P = 0.027) as independent predictor against HBsAg reversion. No ALT flares and adverse events were observed in these HBsAg reverted patients during follow-up, while six patients had detectable HBV DNA.

**Conclusion:**

Low HBsAg and HBeAg negative patients exhibited favorable long-term persistence of HBsAg clearance induced by Peg-IFNα-2b. We identified the EOT HBsAb levels (with levels >1000 IU/mL predicting 100% sustained clearance) and the duration of consolidation therapy as key determinants of long-term treatment success, and proposed 96 weeks as the optimal follow-up time point for these patients.

## 1 Introduction

Currently, global experts recognize HBsAg clearance as the ideal endpoint for patients with chronic hepatitis B (CHB), which can significantly reduce the incidence of hepatocellular carcinoma (HCC) (Yip et al., 2019; Vittal et al., 2022). However, achieving and consolidating this goal in patients at different stages of CHB remains a major clinical challenge.

Spontaneous or nucleos(t)ide analogue (NA) induced HBsAg clearance rarely occurs (Lai et al., 2017; Yip et al., 2017). In contrast, pegylated interferon (Peg-IFN) therapy is superior to NA monotherapy in achieving HBsAg clearance within a limited treatment period, particularly in patients with baseline HBsAg levels <1500 IU/ml, with clearance rates exceeding 30% (Ning et al., 2014; Hu et al., 2018; Wu et al., 2020a). It has been well-documented that baseline HBsAg is one of the strongest predictors of Peg-IFN response (Ning et al., 2014; Jeng et al., 2018; Wen et al., 2023). Evidence from a study indicates that in patients with HBsAg levels <200 IU/ml, a 24-week short-term treatment can achieve an HBsAg clearance rate of 52.1% (Li et al., 2024).

A key concern, however, is long-term durability of HBsAg loss. The persistence of cccDNA and integrated HBV DNA in patients with HBsAg loss creates a risk for HBV reactivation, potentially leading to virological relapse or HBsAg reversion (Tang et al., 2018). Therefore, the long-term durability of HBsAg loss after Peg-IFN discontinuation remains a focus in the field. Recent studies have shown that the sustained HBsAg clearance rate after 48 weeks of follow-up is approximately 80% (Li et al., 2019; Chen et al., 2025). However, these studies enrolled patients with heterogeneous baseline characteristics and lacked research on the durability of HBsAg clearance in advantaged patients with low baseline HBsAg levels.

Hence, we conducted a retrospective study on a cohort of undetectable HBV DNA, HBeAg-negative patients with baseline HBsAg levels <200 IU/mL who achieved HBsAg loss via Peg-IFNα-2b. We aimed to assess the long-term durability of HBsAg loss and to identify factors associated with recurrence. Our findings seek to inform evidence-based strategies for post-treatment monitoring and functional cure management.

## 2 Study design and methods

### 2.1 Patients

In our study, we retrospectively gathered clinical data from patients who obtained HBsAg loss based on Peg-IFNα-2b therapy at The Second Affiliated Hospital of Chongqing Medical University, from July 2019 to June 2025.

Inclusion criteria were as follows: (1) HBsAg positivity for at least 6 months; (2) baseline HBsAg<200IU/ml, HBeAg negativity, HBV DNA undetectable; (3) treatment with Peg-IFNα-2b alone or in combination with NA; (4) achievement of HBsAg clearance (HBsAg<0.05IU/ml) during treatment; (5) regular follow-up every 3 to 6 months post-clearance. Exclusion criteria included: (1) co-infection with other viruses [e.g., human immunodeficiency virus (HIV), hepatitis D virus (HDV), hepatitis C virus (HCV)], liver cancer, alcoholic liver disease, autoimmune hepatitis, or other liver diseases; (2) decompensated cirrhosis, including a history of complications such as gastrointestinal bleeding, ascites, or hepatic encephalopathy; (3) pregnant or breastfeeding women; (4) patients using corticosteroids, immunosuppressants, immunotherapy drugs, or those with a history of alcohol or drug abuse during the follow-up period; (5) patients who did not adhere to regular follow-up. The initial dose of Peg-IFNα-2b for patients was 180 micrograms (mg), administered once weekly. During the course of treatment, dose adjustments were made according to the instructions. Dose reduction should be considered, if absolute neutrophil count (ANC) < 0.75 × 109/L or PLT < 50 × 109/L. Discontinuation of Peg-IFNα-2b was necessary if ANC < 0.50 × 109/L, PLT < 25 × 109/L or serious adverse events (AEs) occurred. The diagnostic criteria for MAFLD (metabolic-associated fatty liver disease) refer to the international expert consensus statement (Eslam et al., 2020). The study complies with good clinical practice and the Declaration of Helsinki and was approved by the Ethics Committee of the Second Affiliated Hospital of Chongqing Medical University.

### 2.2 Definitions

The follow-up period commenced upon discontinuation of Peg-IFNα-2b therapy and extended until the HBsAg reversion. Patients who did not experience HBsAg reversion would continue to be observed for any potential changes. HBsAg clearance was defined as a serological conversion to negative HBsAg (HBsAg < 0.05 IU/mL) following PegIFNα-2b treatment. HBsAg sustained response was defined as sustained HBsAg loss with or without the appearance of HBsAb at the end of follow-up (EOF). HBsAg reversion was defined as the reappearence of HBsAg (≥ 0.05 IU/mL) during follow-up after an initial conversion to negative. Virologic reversion was defined as HBV DNA level returning to ≥20 IU/ml.

### 2.3 Laboratory measurements

Serum HBsAg, anti-HBs and HBeAg was measured using Elecsys HBsAg II quant II, Elecsys Anti-HBs II, and Elecsys HBeAg (Roche Diagnostics GmbH, Mannheim, Germany), respectively. HBsAg negativity was set at HBsAg levels < 0.05 IU/mL, and anti-HBs positivity was set at anti-HBs levels ≥ 10 IU/L. Serum HBV DNA was detected using the TaqMan-based real-time polymerase chain reaction assay (Shanghai ZJ BioTech, Shanghai, China) with a limit of detection of 20IU/ml. Serum ALT was assayed using an automatic biochemical analyzer (Roche, Basel, Switzerland) and presented as multiples of the upper limit of normal value (ULN) (men, 50 IU/L; women, 40 IU/L).

### 2.4 Statistical analysis

The analyses were performed using the Statistical Package for Social Sciences (SPSS, version 26.0, Chicago, IL, USA) and Graph Pad Prism version 9.5.0, with two-tailed tests at a significance level of 0.05. Based on the results of the Kolmogorov–Smirnov normality test, normal distribution variables are expressed as means ± standard deviations, and non-normal distribution continuous variables are expressed as median and interquartile ranges (Q1–Q3). Categorical variables are reported as counts and percentages. To avoid immortal time bias, we used a time−varying covariate Cox model for consolidation therapy. Correlations between continuous variables were analyzed using Spearman’s rank correlation. Differences from the baseline characteristics and treatment data were compared using a χ^2^ test for categorical variables or the one-way ANOVA for continuous variables. The Kaplan–Meier method estimated the cumulative rates, followed by a Log-rank test to compare the differences between the different groups. Cox regression analysis was performed to evaluate the indicators associated with HBsAg reversion.

## 3 Results

### 3.1 Clinical characteristic of enrolled patients

**Figure 1:**
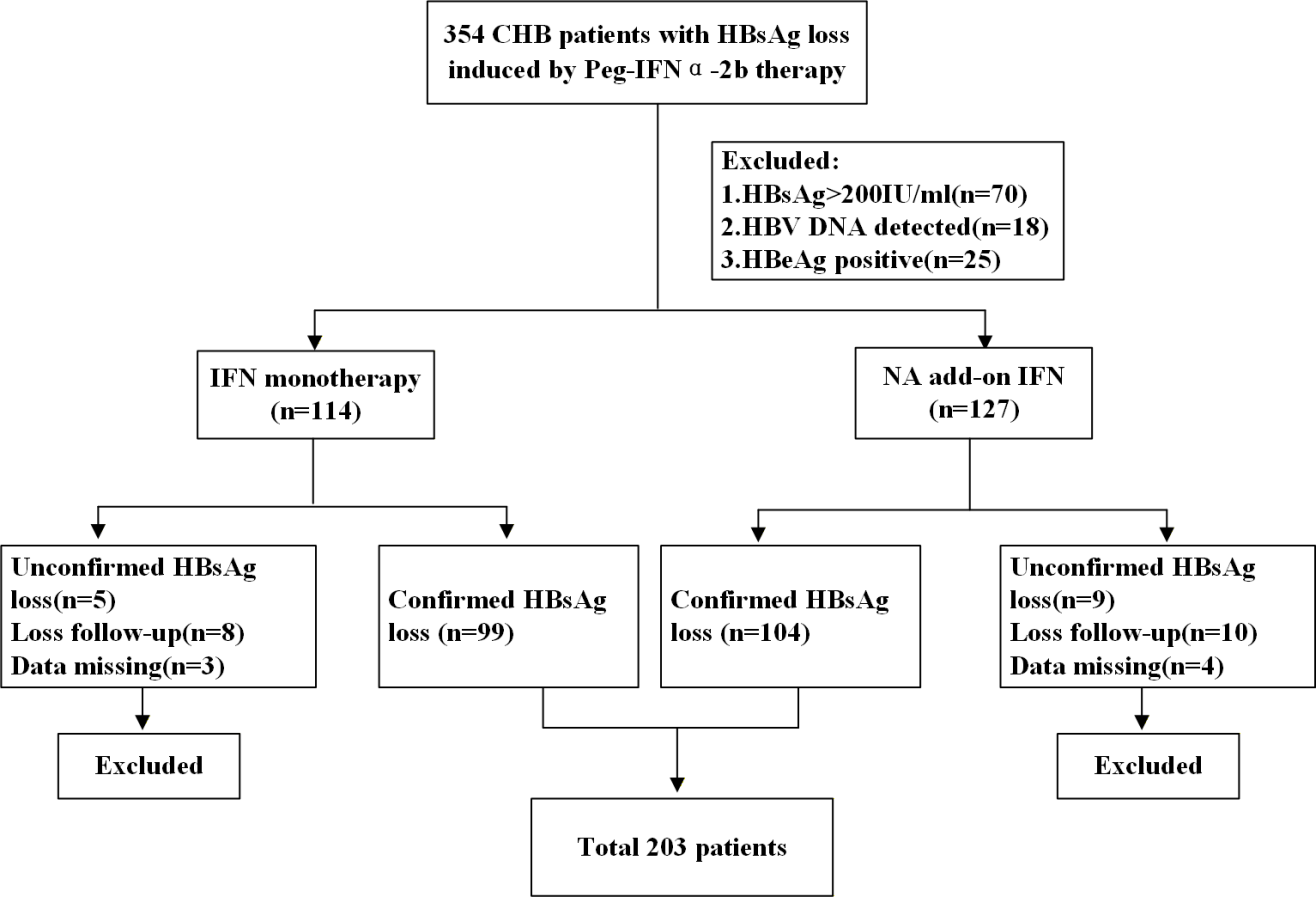
Flow diagram of patients’ screening process. After screening, a total of 203 patients were included for clinical analysis. CHB, Chronic hepatitis B; Peg-IFNα-2b, Pegylated interferon α-2b; HBsAg, Hepatitis B surface antigen; NA, nucleos(t)ide analog.

We enrolled 354 patients with HBsAg loss induced by Peg-IFNα-2b. After excluding patients with baseline HBsAg >200 IU/mL (n=70), detectable HBV DNA (n=18), and positive HBeAg (n=25), there were 114 patients received Peg-IFNα-2b monotherapy and 127 patients received NA combination therapy, respectively. Subsequently, we further excluded patients who were lost to follow-up, had missing data, or had uncertain HBsAg clearance status. Ultimately, a total of 203 patients were included in this study. The flow chart illustrating case screening was presented in **Figure 1**.

The mean age was 42.480 ± 8.534 years old, with 136 males (67.000%), 41 patients concurrent MAFLD and 99 patients received Peg-IFNα-2b monotherapy. The median (IQR) follow-up period after Peg-IFNα-2b discontinuation was 96 (72-120) weeks, with a maximum follow-up of 276 weeks. Among the 203 patients, 44 developed HBsAg reversion during the follow-up, yielding a HBsAg reversion rate of 21.670% (44/203). The HBsAg reversion rate during follow-up was similar between Peg-IFN monotherapy group and NA add-on Peg-IFN group (24.242% VS 19.231%, P = 0.321) (**Table 1**). As shown in **Supplementary Table 1**, the two groups were comparable in most other baseline characteristics.

**Table 1:** Baseline characteristics and study summary of patients with sustained HBsAg clearance versus HBsAg reversion.

Characteristics	All	Sustained HBsAg loss	HBsAg reversion	P value
	203	159 (78.330%)	44 (21.670%)	
Age, years (Mean ± SD)	42.480 ± 8.534	43.000 ± 8.742	40.590 ± 7.531	**0.011**
Sex, male (n, %)	136 (67.000%)	108 (79.400%)	28 (20.600%)	0.515
Treat regimens				0.312
Peg-IFN (n, %)	99 (48.770%)	75 (75.760%)	24 (24.240%)	
NA add-on Peg-IFN (n, %)	104 (51.230%)	84 (80.770%)	20 (19.230%)	
Combined NAs (n=104)				0.545
ETV (n, %)	56 (53.850%)	47 (55.952%)	9 (45.000%)	
TDF (n, %)	26 (24.040%)	21 (25.000%)	5 (25.000%)	
TAF (n, %)	22 (22.110%)	16 (19.048%)	6 (30.000%)	
HBsAg, IU/ml (Mean ± SD)	41.877 ± 3.467	35.868 ± 3.639	63.591 ± 8.406	**0.001**
HBsAg				0.011
<100 (n, %)	180 (88.670%)	147 (92.453%)	33 (75.000%)	
100-200 (n, %)	23 (11.330%)	12 (7.547%)	11 (25.000%)	
HBeAg negative (n, %)	203 (100.000%)	159 (100.000%)	44 (100.000%)	1.000
HBV DNA negative (n, %)	203 (100.000%)	159 (100.000%)	44 (100.000%)	1.000
Alb, g/L, Median (Q1-Q3)	47.100 (44.900, 49.000)	47.000 (44.700, 48.900)	47.400 (45.725, 49.790)	0.268
ALT, U/L, Median (Q1-Q3)	23.000 (16.800, 32.000)	22.000 (17.000, 32.000)	24.300 (16.000, 29.675)	0.325
AST, U/L, Median (Q1-Q3)	23.000 (19.000, 27.000)	23.000 (19.000, 27.000)	21.525 (18.835, 26.270)	0.222
ALP, U/L, Median (Q1-Q3)	66.000 (56.000, 75.500)	67.000 (58.000, 76.000)	65.000 (53.000, 73.000)	0.841
GGT, U/L, Median (Q1-Q3)	19.000 (15.000, 28.000)	18.600 (15.000, 26.600)	20.600 (16.000, 32.375)	**0.018**
TB, μmol/L, Median (Q1-Q3)	12.100 (9.100, 15.960)	12.000 (8.700, 15.930)	12.160 (9.850, 16.236)	0.608
RBC, 10^9^/L, Median (Q1-Q3)	4.980 (4.580, 5.340)	4.980 (4.580, 5.320)	4.950 (4.565, 5.402)	0.837
WBC, 10^9^/L, Median (Q1-Q3)	5.640 (4.780, 6.800)	5.720 (4.780, 6.820)	5.465 (4.780, 6.438)	**0.022**
PLT, 10^9^/L, Median (Q1-Q3)	202 (170, 236)	201 (167, 228)	216.5 (184.5, 252.5)	**<0.001**
AFP, μg/L, Median (Q1-Q3)	2.510 (1.900, 3.400)	2.500 (1.890, 3.360)	2.800 (2.000, 4.020)	0.117
MAFLD (n, %)	41 (20.200%)	29 (70.730%)	12 (29.270%)	0.157
Fibroscan scores, Kpa, Median (Q1-Q3)	5.600 (4.500, 6.400)	5.500 (4.600, 6.300)	5.700 (4.500, 6.800)	0.177
HBcAb^a^, IU/ml, Median (Q1-Q3)	6.990 (6.270-7.820)	6.910 (6.120, 7.810)	7.390 (6.690, 8.080)	**0.001**
HBsAb^a^, IU/ml, Median (Q1-Q3)				<0.001
<100 (n, %)	113 (55.665%)	74 (46.541%)	39 (88.636%)	
≥100 (n, %)	100 (49.261%)	85 (53.459%)	5 (11.364%)	
Consolidation therapy, week, Median (Q1-Q3)	12 (8, 24)	12 (12, 24)	8 (0, 12)	**<0.001**
Consolidation therapy				<0.001
<12 weeks (n, %)	52 (13.800%)	29 (18.240%)	23 (52.273%)	
≥12 weeks (n, %)	151 (32.00%)	130 (81.760%)	21 (47.727%)	
Therapy duration, wk, Median (Q1-Q3)	36 (24, 48)	36 (24, 48)	32 (24, 48)	0.701
Therapy time before HBsAg loss, wk, Median (Q1-Q3)	16 (12, 24)	12 (12, 24)	24 (16, 36)	**<0.001**
Follow-up time, wk, Median (Q1-Q3)	96 (72-120)	96 (68, 120)	96 (82, 138)	0.272

HBcAb^a^, HBcAb levels at end of Peg-IFNα-2b treatment; HBsAb^a^, HBsAb levels at end of Peg-IFNα-2b treatment; Consolidation treatment, continuing therapy by pegylated interferonα-2b after HBsAg loss. SD, standard deviation; TDF, tenofovir disoproxil fumarate; ETV, entecavir; TAF, tenofovir alafenamide; HBeAg, hepatitis B e-antigen; HBsAg, hepatitis B surface antigen; HBV DNA, hepatitis B virus-deoxyribonucleic acid; ALT, alanine aminotransferase; AST, aspartate aminotransferase; ALP, alkaline phosphatase; GGT, gamma-glutamyl tanspepetidase; RBC, red blood cell; WBC, white blood cell; TB, total bilirubin; A, albumin; PLT, platelets; AFP, alpha-fetoprotein; Peg-IFN, pegylated interferon α-2b; NA, nucleoside analog; HBsAb, hepatitis B surface antigen; HBcAb, hepatitis B core antigen; IQR, interquartile range; EOT, end of Peg-IFNα-2b treatment. MAFLD, Metabolic associated fatty liver disease. The values marked in bold signify statistical significance (p<0.05).

In patients with sustained HBsAg loss, 53.459% HBsAb > 100IU/ml at EOT, while this percentage was 11.364% in those with HBsAg reversion (P < 0.001). Baseline HBsAg level were significantly lower in the sustained HBsAg clearance group than HBsAg reversion group(P = 0.001). Additionally, patients with sustained HBsAg clearance received longer consolidation therapy and achieved clearance more rapidly than those who developed HBsAg reversion (both P<0.001). There were significant differences between the two groups in terms of baseline GGT, WBC, PLT and EOT HBcAb levels. Furthermore, no significant differences were observed between the two groups regarding sex, types of NAs used, treatment regimens, presence of MAFLD, baseline ALT, AST, Alb, TB, ALP, RBC, AFP, Fibroscan scores, Peg-IFN therapy duration and follow-up time (**Table 1**).

### 3.2 Distribution of HBsAg reversion time

**Figure 2:**
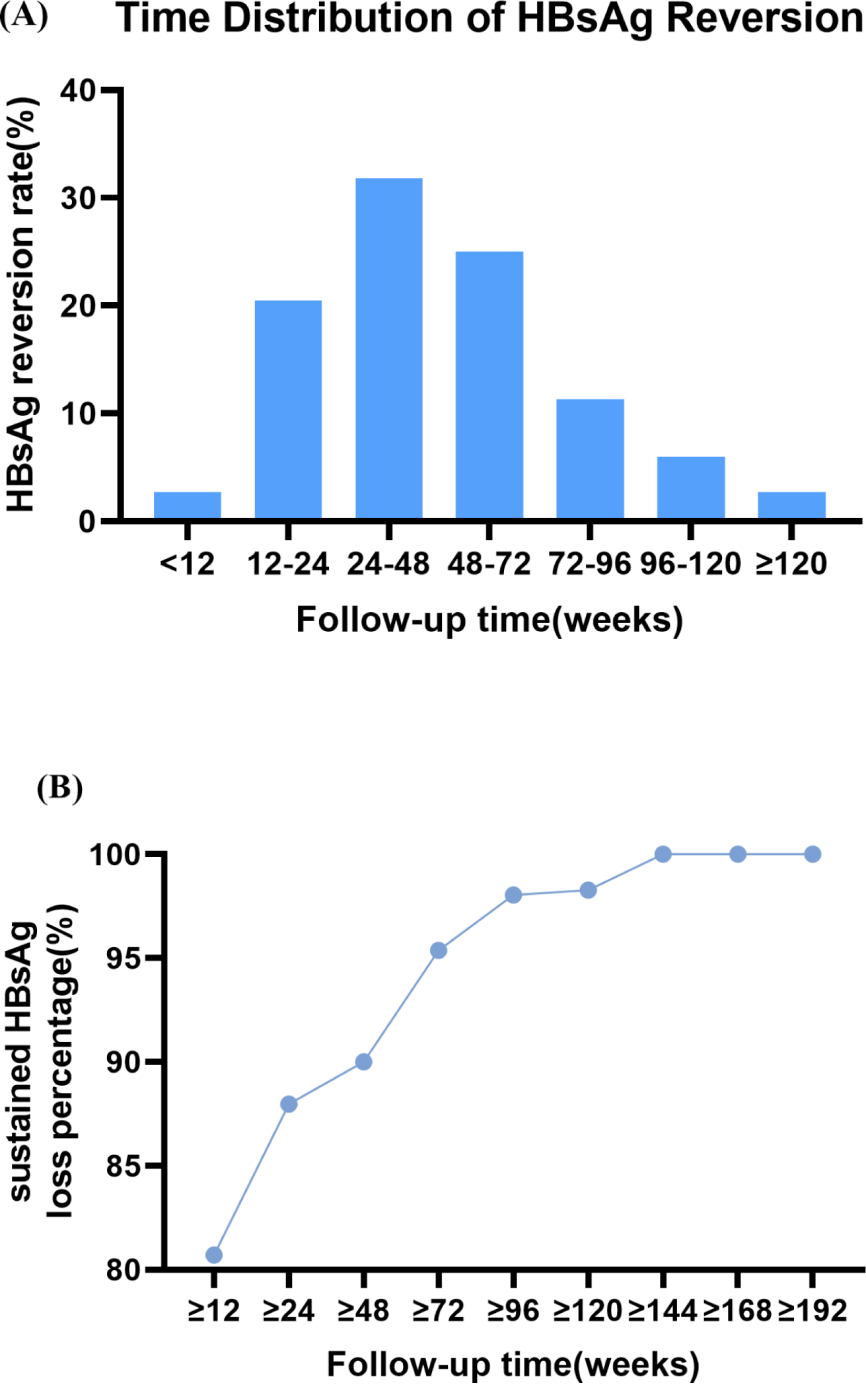
**(A)** Time distribution of HBsAg reversion. **(B)** Trend of sustained HBsAg loss percentage with follow-Up duration.

Among the 44 patients with HBsAg reversion, ten (23.18%) patients experienced recurrence within 24 weeks after Peg-IFNα-2b discontinuation. The remaining cases recurred at later intervals: fourteen (31.82%) between 24–48 weeks, eleven (25%) between 48–72 weeks, five (11.36%) between 72–96 weeks, three (5.92%) between 96–120 weeks, and one (2.72%) after 120 weeks. The cumulative HBsAg recurrence rate was 55% (24/44) within 48 weeks after treatment discontinuation and increased to 91.36% (40/44) by 96 weeks (**Figure 2A**; **Supplementary Table 2**). Correspondingly, the sustained HBsAg clearance percentage within 96 weeks was 98.04% (100/102). Hence, given that the majority of recurrences occurred within the first 96 weeks, this period could be recommended as the ideal follow-up interval for patients with low baseline HBsAg who achieve clearance via Peg-IFNα-2b therapy (**Figure 2B**; **Supplementary Table 3**).

### 3.3 Comparison of sustained HBsAg clearance between different subgroups

**Figure 3:**
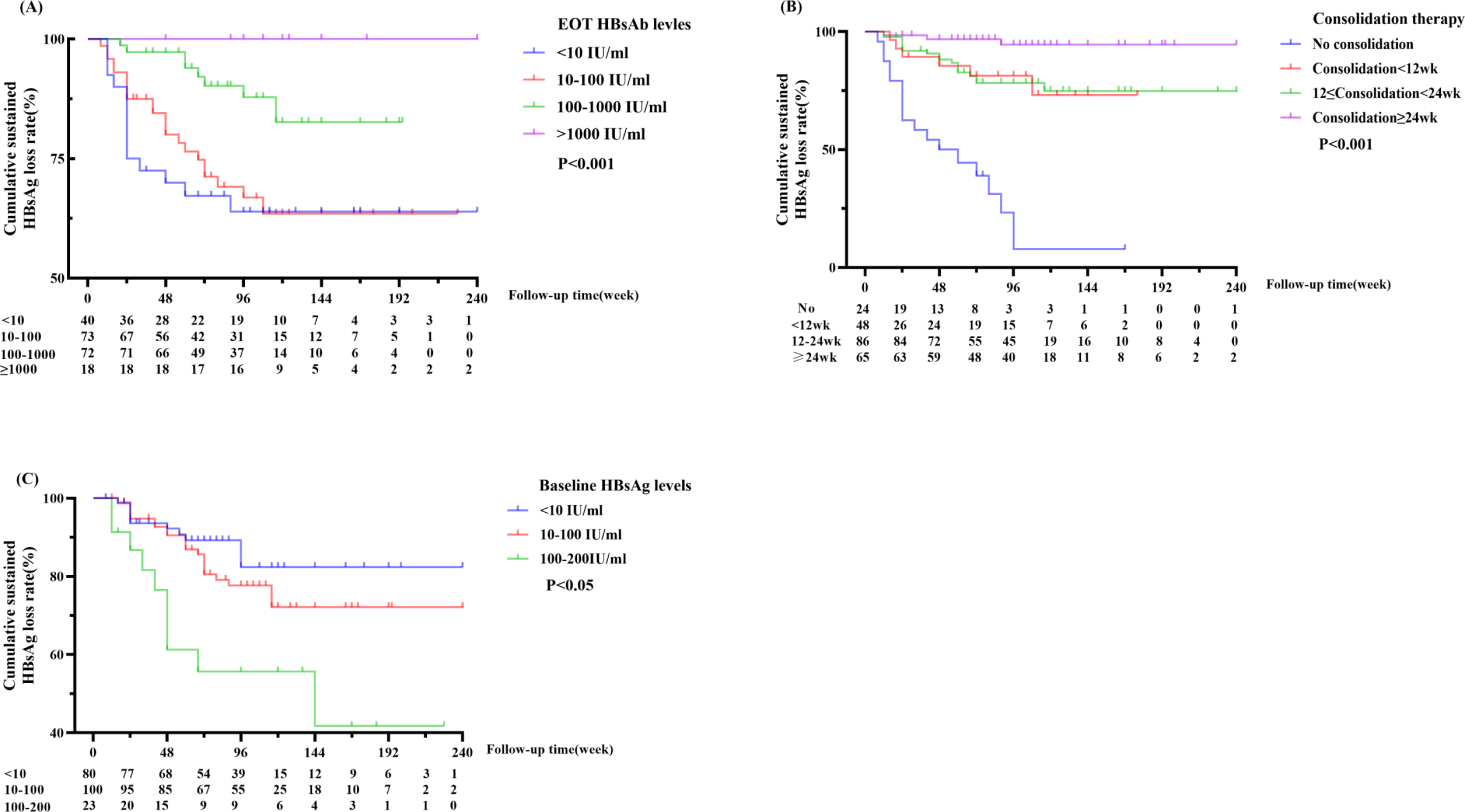
**(A)** Cumulative sustained HBsAg loss rate stratified by EOT HBsAb levels over follow-up time. **(B)** Impact of consolidation therapy duration on cumulative sustained HBsAg loss rate during follow-up. **(C)** Comparison of cumulative sustained HBsAg loss rate by different baseline HBsAg levels over follow-up.

Kaplan-Meier analysis revealed a significant difference in the sustained HBsAg clearance rate among groups stratified by HBsAb levels at EOT (P < 0.001). Sustained HBsAg clearance rates showed a gradual increase with rising EOT HBsAb titers. Specifically, the group with HBsAb ≥1000 IU/mL achieved a 100% sustained response rate during the follow-up, whereas HBsAb negative group had a rate of only63.860% at 96 weeks. (**Figure 3A**).

Next, to determine whether consolidation therapy contributes to sustained HBsAg loss, patients were stratified into four groups: no consolidation, consolidation therapy <12 weeks, 12–24 weeks, and ≥24 weeks. **Figure 3B** shows a clear positive correlation: longer consolidation therapy is associated with a higher sustained HBsAg loss rate. The group with consolidation therapy ≥24 weeks achieved the highest and most stable rate, remaining at 94.490% at 96-week follow-up. In contrast, the no consolidation group exhibited a rapid decline in durable HBsAg loss rate, approaching 7.770% at 96 weeks. The difference among the four groups was statistically significant (P<0.001), confirming that the duration of consolidation therapy is a critical factor in maintaining HBsAg clearance.

Previous studies have indicated that baseline HBsAg level is a predictor of HBsAg loss, yet its influence on the durability of pose-treatment HBsAg loss remains unclear. A significant inverse relationship was observed between different baseline HBsAg levels and the sustained HBsAg loss rate (P < 0.05). Patients in the lowest HBsAg group (HBsAg <10 IU/ml) had the highest probability of maintaining sustained HBsAg clearance, with the rate remaining stable at 82.388% at 96-week follow-up. In contrast, the intermediate group (10–100 IU/ml) exhibited a moderate decline in clearance rate, while the highest group (100–200 IU/ml) demonstrated the poorest sustained response, with the rate dropping to 41.746% at 144 weeks.

### 3.4 Correlation analysis of HBsAb levels with clinical and virological parameters

**Figure 4:**
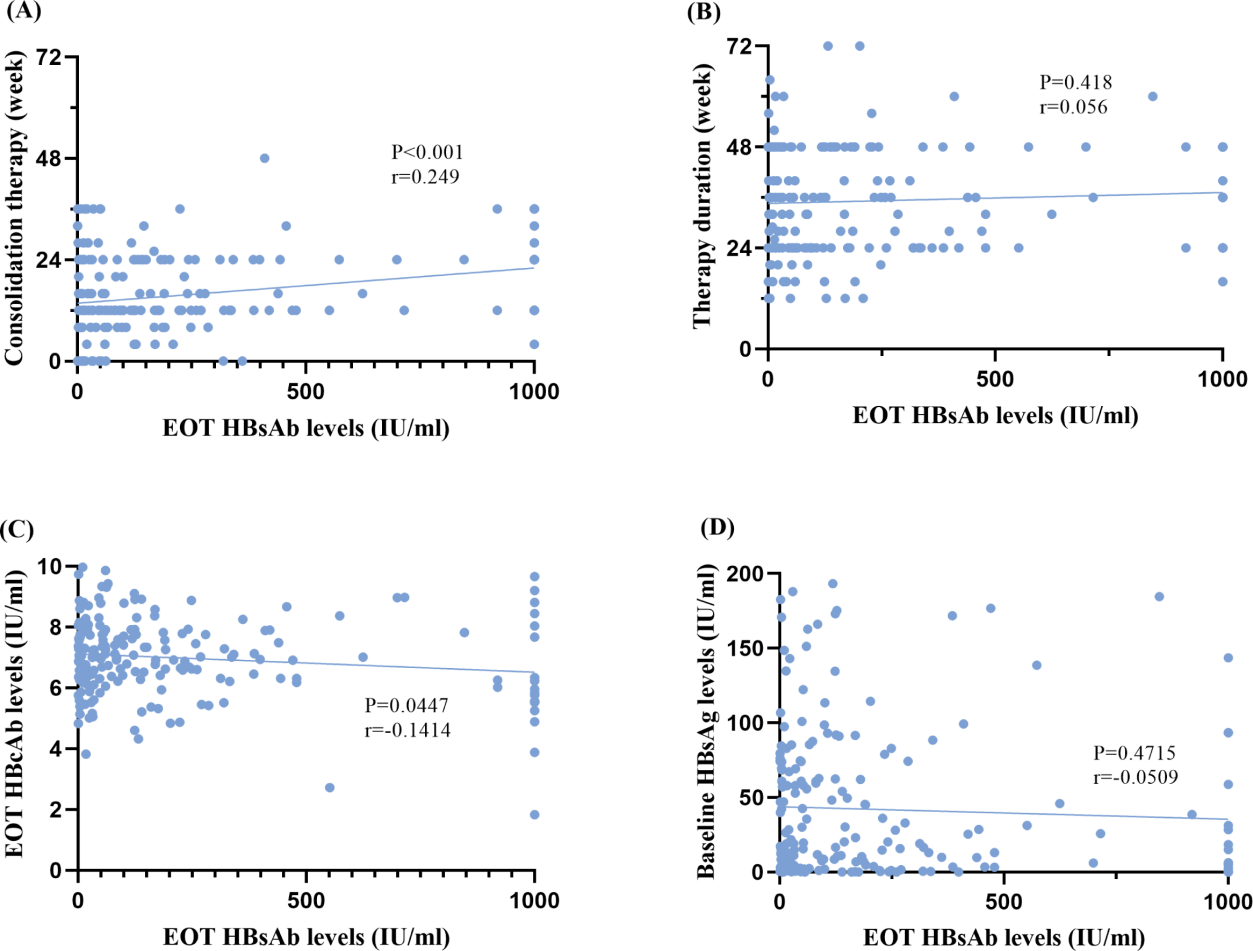
**(A)** Correlation between end-of-treatment HBsAb levels and consolidation therapy duration. **(B)** Association between end-of-treatment HBsAb levels and Peg-IFNα-2b therapy duration. **(C)** Correlation between end-of-treatment HBsAb levels and end-of-treatment HBcAb levels. **(D)** Relationship between end-of-treatment HBsAb levels and baseline HBsAg levels.

**Figure 4** illustrates the correlation analysis between EOT HBsAb levels and both virological parameters and Peg-IFNα-2b treatment parameters. We found that EOT HBsAb levels showed a positive correlation with the consolidation treatment duration (P<0.001, r=0.249) (**Figure 4A**), but no significant correlation with the total Peg-IFNα-2b treatment duration (P = 0.418, r=0.056) (**Figure 4B**), indicating that consolidation treatment duration, rather than total treatment duration, is the key modifiable factor modulating the strength of the humoral immune response. For virological markers, HBsAb levels at EOT were negatively correlated with EOT HBcAb levels (P = 0.045, r=-0.141) (**Figure 4C**), whereas no significant correlation was observed between EOT HBsAb levels with baseline HBsAg levels (P = 0.472, r=-0.051) (**Figure 4D**).

### 3.5 Risk factor for HBsAg reversion

Age, gender, treatment regimens, combined NA, baseline HBsAg levels, EOT HBsAb levels, HBcAb levels, baseline GGT, WBC, PLT, Alb, ALT, AST, RBC, TB, FibroScan scores, AFP, MAFLD, and Peg-IFNα-2b therapy duration were included as covariates to develop a Cox proportional hazards model. To avoid immortal time bias, consolidation therapy was modeled as a time−varying covariate. Univariate analysis indicated baseline HBsAg levels (HR = 0.459, 95%CI =0.226–0.933, P = 0.031), EOT HBsAb levels (HR = 0.185, 95%CI =0.073–0.471, P<0.001), consolidation treatment duration (HR = 0.316, 95%CI =0.174–0.572, P<0.001) and baseline WBC (HR = 0.790, 95%CI =0.625–0.999, P = 0.049) were significantly associated with HBsAg reversion. Multivariate analysis showed that HBsAb levels at EOT ≥100 IU/ml (HR = 0.238, 95%CI =0.092–0.617, P = 0.003) and consolidation treatment ≥12 weeks (HR = 0.492, 95%CI =0.263–0.921, P = 0.027) as independent predictive factors against HBsAg reversion (**Table 2**).

**Table 2:** Cox regression analysis of factors associated with HBsAg reversion.

Characteristics	Statistic (%)	Univariable (HR, 95% CI)	P value	Multivariable (HR, 95% CI)	P value
**Age (Mean ± SD)**	42.480 ± 8.534	0.966 (0.931, 1.002)	0.063		
Gender (n, %)					
Male	136 (67.000%)	1			
Female	67 (33.000%)	1.550 (0.826, 2.907)	0.172		
Treatment regimens (n, %)					
Peg-IFN	99 (48.770%)	1			
NA add-on Peg-IFN	104 (51.230%)	0.74 (0.407, 1.345)	0.323		
Combined NA (n, %)					
ETV	56 (53.850%)	1			
TDF	25 (24.040%)	1.479 (0.494, 4.427)	0.484		
TAF	23 (22.110%)	2.439 (0.865, 6.873)	0.092		
HBsAg^a^ (n, %)					
100-200IU/ml	180 (88.670%)	1		1	
<100IU/ml	23 (11.330%)	0.459 (0.226, 0.933)	**0.031**	0.579 (0.273, 1.229)	0.155
HBsAb^a^ (n, %)					
<100IU/ml	113 (55.665%)	1		1	
>100IU/ml	90 (44.335%)	0.185 (0.073, 0.471)	**<0.001**	0.238 (0.092, 0.617)	**0.003**
Consolidation treatment (n, %)					
<12 weeks	52 (25.616%)	1		1	
≥12 weeks	151 (74.384%)	0.316 (0.174, 0.572)	**<0.001**	0.492 (0.263, 0.921)	**0.027**
MAFLD (n, %)					
YES	41 (20.200%)	1			
NO	162 (79.800%)	0.933 (0.478, 1.820)	0.839		
**GGT, U/L**	19 (15, 28)	1.010 (0.997, 1.023)	0.126		
**WBC, 10^9^/L**	5.640 (4.780, 6.800)	0.790 (0.625, 0.999)	**0.049**	0.857 (0.663, 1.109)	0.241
**PLT, 10^9^/L**	202 (170, 236)	1.004 (0.999, 1.009)	0.135		
**Fibroscan scores, Kpa**	5.600 (4.500, 6.400)	0.955 (0.806, 1.132)	0.597		
**Alb, g/L**	47.100 (44.900, 49.000)	1.075 (1.000, 1, 158)	0.051		
**ALT, U/L**	23.000 (16.800, 32.000)	0.984 (0.958, 1.012)	0.263		
**AST, U/L**	23.000 (19.000, 27.000)	1 (0.968, 1.033)	0.983		
**RBC, 10^9^/L**	4.960 (4.580, 5.320)	0.778 (0.437, 1.385)	0.394		
**TB, μmol/L**	12.100 (9.100, 15.960)	1.018 (0.971, 1.067)	0.457		
**Therapy duration, week**	36 (24, 48)	0.997 (0.973, 1.021)	0.786		
**HBcAb^a^**	6.990 (6.270, 7.820)	1.170 (0.937, 1.461)	0.165		
**AFP, μg/L**	2.510 (1.900, 3.400)	1.126 (0.953, 1.332)	0.161		

HBsAg^a^, baseline HBsAg levels; HBcAb^a^, HBcAb levels at end of Peg-IFNα-2b treatment; HBsAb^a^, HBsAb levels at end of Peg-IFNα-2b treatment; Consolidation treatment, continuing therapy by pegylated interferonα-2b after HBsAg loss. HR, hazard ratio; CI, confidence interval; TDF, tenofovir disoproxil fumarate; ETV, entecavir; TAF, tenofovir alafenamide; HBsAg, hepatitis B surface antigen; ALT, alanine aminotransferase; AST, aspartate aminotransferase; ALP, alkaline phosphatase; GGT, gamma-glutamyl tanspepetidase; WBC, white blood cell; TB, total bilirubin; Alb, albumin; PLT, platelets; AFP, alpha-fetoprotein; Peg-IFN, Pegylated interferonα-2b; NA, nucleoside analog; HBsAb, hepatitis B surface antigen; HBcAb, hepatitis B core antigen; MAFLD, Metabolic associated fatty liver disease. The values marked in bold signify statistical significance (p<0.05).

### 3.6 Safety analysis in patients with HBsAg reversion

Among the 44 patients with HBsAg reversion, the mean HBsAg and HBsAb levels at recurrence were 1.01 IU/mL and 6.62 IU/mL, respectively. The median (IQR) ALT and AST levels were 23 (15.25–29.00) U/L and 23 (19.00–27.75) U/L. Of these patients, six had concurrently detectable HBV DNA (one patient HBV DNA level is 233.11 IU/ml, the other patients below 100 IU/ml). No patient experienced ALT flare. During follow-up, no adverse events such as liver failure, decompensated cirrhosis, or hepatocellular carcinoma were observed in the patients with HBsAg reversion.

Among the 20 patients who experienced reversion after NA combined with Peg-IFNα-2b therapy, six opted for retreatment with the same combination regimen and all achieved a second HBsAg clearance. The remaining patients continued on NA monotherapy. Of the 23 recurrence cases initially treated with Peg-IFNα-2b monotherapy, four were retreated with Peg-IFNα-2b and regained HBsAg clearance, two initiated NA therapy due to detectable HBV DNA, and the rest were maintained on close follow up every 3–6 months (**Supplementary Table 4**).

## 4 Discussion

In chronic HBV infection, prolonged exposure to viral antigens leads to functional suppression of dendritic cells (DCs), natural killer (NK) cells, and T cells, making it difficult for the body to spontaneously recover from the infection (Chen et al., 2005; Yu et al., 2010; Hatipoglu et al., 2014; Ye et al., 2015). Nucleos(t)ide analogs (NAs) exert direct antiviral effects but fail to reverse the immune suppression, resulting in a markedly low rate of HBsAg clearance (Lok et al., 2020). NA discontinuation in patients with HBsAg <200 IU/ml can achieve an HBsAg clearance rate of approximately 25%, yet this strategy carries a risk of hepatic inflammatory flares (Liu et al., 2019; Fan et al., 2025). In contrast, pegylated interferon (Peg-IFN) not only exerts robust immunomodulatory effects, restoring host immune function by activating innate immunity and reshaping HBV-specific T-cell and B-cell responses (Micco et al., 2013), but also has antiviral effects (Lei et al., 2024). Therefore, Peg-IFN serves as the cornerstone for functional cure of chronic HBV infection, particularly in HBeAg-negative patients with low baseline HBsAg levels, who are more likely to achieve HBsAg clearance with Peg-IFN therapy (Ning et al., 2014; Jeng et al., 2018; Wen et al., 2023). Consequently, the majority of patients selected for Peg-IFN treatment in clinical practice are those with HBsAg <200 IU/ml, HBeAg negative status, and undetectable HBV DNA.

Besides Peg-IFN, bepirovirsen—an antisense oligonucleotide targeting all HBV RNA transcripts—has demonstrated promising HBsAg clearance in clinical trials. However, 75% of participants experienced relapse after treatment cessation (Yuen et al., 2022). Similarly, due to the inability to completely eliminate intrahepatic cccDNA and integrated HBV DNA, some patients still experienced HBsAg reversion even after achieving HBsAg clearance with Peg-IFN therapy (Gao et al., 2023). Wu et al (Wu et al., 2020b). reported an HBsAg reversion rate of 9.66% after a median follow-up of 160 weeks. Li et al (Li et al., 2019). observed an HBsAg recurrence rate of 12.79% at 48 weeks post-treatment, while Lok et al (Lok et al., 2020). reported an 18% HBsAg recurrence rate at 96 weeks. Building upon the finding of previous studies, our research further investigates the sustainability of HBsAg loss in advantaged patients with low HBsAg levels, HBeAg-negative and undetectable HBV DNA in the real-world.

In our cohort, an HBsAg relapse rate of 21.67% (44/203) was observed during a median follow-up of 96 weeks, which is close to the 21.41% rate reported by Chen et al (Chen et al., 2025). Despite the inclusion of patients with even lower baseline HBsAg levels, our relapse rate was higher than those reported in the studies by Li and Wu (Li et al., 2019; Wu et al., 2020b). This discrepancy may reflect differences in host immune backgrounds in real-world clinical settings, and could also be related to variations in HBsAg detection assays. Nevertheless, our results also confirm that the majority of patients with low HBsAg levels were able to maintain HBsAg loss.

Currently, there is no clear consensus on the optimal follow-up interval after HBsAg clearance, some studies have identified 24 weeks as an appropriate follow-up duration (Yip and Lok, 2020), yet reversion events often occur beyond this timeframe (Li et al., 2019; Wang et al., 2025). In this study, 55% (24/44) of HBsAg recurrences occurred within 48 weeks of follow-up, with a peak incidence noted between 24 and 48 weeks. Therefore, monitoring every 12 weeks during the first 48 weeks after treatment cessation is recommended. Few patients experienced HBsAg relapse after 96 weeks, which is consistent with evidence demonstrating reduced intrahepatic covalently closed circular DNA (cccDNA) and integrated HBV DNA levels in long-term responders (Li et al., 2019; Gao et al., 2023), suggesting that 96 weeks may serve as an ideal follow-up interval.

Some patients still experienced relapse after Peg-IFN discontinuation. Therefore, identifying the risk factors of relapse is of particular importance for clinical treatment decision-making. Our study enrolled patients with low HBsAg levels and univariate Cox regression analysis revealed fewer patients with sustained response in the 100–200 IU/mL HBsAg subgroup (HR = 0.459, P = 0.031), whereas baseline HBsAg level was not a key determinant of the final immune response in the multivariate Cox regression model (HR = 0.501, P = 0.163). Previous studies have reported an association between EOT HBsAb levels> 100 IU/mL and sustained response (Wu et al., 2020b; Huang et al., 2022), while Chen et al (Chen et al., 2025). identified HBsAb > 25.67 IU/mL as a predictive factor for sustained HBsAg clearance. A trend of increasing sustained response rate was observed with elevation of HBsAb levels across patient subgroups(P<0.001), and no patients in the HBsAb > 1000 IU/mL subgroup experienced HBsAg reversion. Our study indicates that patients with EOT HBsAb >1000 IU/mL rarely experienced HBsAg reversion, achieving a sustained HBsAg clearance rate of 100%. Multivariate Cox regression analysis confirmed that HBsAb ≥ 100 IU/mL was a significant independent predictive factor against HBsAg relapse (HR = 0.238, P = 0.003). Consolidation therapy after HBsAg clearance also plays pivotal role in sustained HBsAg response. A study has identified 12.786 weeks of consolidation therapy as a predictive factor for sustained HBsAg loss (Chen et al., 2025), and our study also demonstrated a significant association between consolidation therapy exceeding 12 weeks and sustained HBsAg clearance(HR = 0. 492, P = 0.027). Stratification analysis showed that the 96-week sustained HBsAg clearance rate was only 7.77% in patients without consolidation therapy, whereas this rate remained above 90% at 96-week follow-up in patients who received ≥ 24 weeks of consolidation therapy (P < 0.001). This indicates that a longer duration of consolidation therapy is associated with a higher rate of sustained HBsAg clearance.

HBsAb, a product of humoral immunity, is associated with robust immune control of HBV infection (van Campenhout and Janssen, 2015). Furthermore, HBsAb can neutralize circulating HBsAg, which facilitates the achievement of sustained HBsAg clearance. Our study found a negative correlation between EOT HBsAb levels and HBcAb levels, which may suggest that high HBcAb levels impede the immune system from shifting to the production of potent, neutralizing HBsAb under the modulation of Peg-IFN. Meanwhile, our study revealed a positive correlation between HBsAb levels at EOT and the duration of consolidation therapy, with no association observed with Peg-IFN treatment duration. This indicates that the consolidation therapy, rather than the overall treatment course, is the critical period for maintaining HBsAb (anti-HBs) levels. The enhancement of sustained response by consolidation therapy may be achieved by elevating HBsAb levels at treatment discontinuation—a mechanism that may involve persistent immune stimulation promoting the differentiation of B cells into long-lived memory B cells or plasma cells, thereby sustaining antibody levels. The specific underlying mechanisms warrant further investigation.

Most patients with HBsAg reversion following treatment with Peg-IFN and NAs exhibit clinically stable (Chi et al., 2017; Li et al., 2019; Lok et al., 2020). In our study, ALT and AST levels remained within the normal range in all 44 patients with HBsAg reversion, among whom 38 cases (38/44, 86.4%) had undetectable HBV DNA. Of these patients, 10 underwent retreatment with Peg-IFNα-2b, and 100% (10/10) achieved a second HBsAg clearance within 24 weeks of retreatment. This suggests that for patients who experience HBsAg recurrence following Peg-IFN–based therapy, reinitiating Peg-IFN treatment represents a highly effective strategy.

This study also has several limitations. First, its retrospective design carries the potential for inherent selection and data biases, as all enrolled patients were advantageous patients with low baseline HBsAg levels. In addition, the retrospective nature may lead to underreporting of mild or asymptomatic adverse events. Second, several novel biomarkers such as HBV RNA, HBcrAg, which may be associated with sustained response, were not assessed in the study. Third, this is a single-center study, had relatively short follow-up duration. Multicenter studies with larger sample sizes and long observation are needed to validate the current findings.

In conclusion, advantaged patients with low HBsAg levels and HBeAg-negative exhibit a relatively high rate of sustained HBsAg clearance following Peg-IFNα-2b therapy, yet they remain face a risk of relapse, which commonly occurs within 96 weeks after treatment discontinuation. Furthermore, EOT HBsAb levels≥100 IU/mL and consolidation treatment ≥12 weeks are associated with sustained HBsAg clearance in this cohort. Especially, patients with end-of-treatment HBsAb >1000 IU/mL achieved a 100% sustained HBsAg clearance rate, with no HBsAg reversion observed. Notably, consolidation therapy can exert the advantages of stabilizing HBsAb levels and reducing the likelihood of HBsAg reversion. Finally, reinitiating Peg-IFNα-2b therapy constitutes an effective retreatment strategy for patients with HBsAg reversion.

## Data Availability

The raw data supporting the conclusions of this article will be made available by the authors, without undue reservation.

## References

[B1] Chen J. Huang Z. Yang X. Yang Y. Tan M. Duan L. . (2025). Predictors of durable hepatitis B surface antigen loss after pegylated interferon–based therapy in patients with hepatitis B e antigen–negative chronic hepatitis B: A multicenter real-world study. J. Infect. Dis. 232, 953–960. doi: 10.1093/infdis/jiaf198. PMID: 40272911

[B2] Chen Y. Wei H. Sun R. Tian Z. (2005). Impaired function of hepatic natural killer cells from murine chronic HBsAg carriers. Int. Immunopharmacol. 5, 1839–1852. doi: 10.1016/j.intimp.2005.06.004. PMID: 16275620

[B3] Chi H. Wong D. Peng J. Cao J. Van Hees S. Vanwolleghem T. . (2017). Durability of response after hepatitis B surface antigen seroclearance during nucleos(t)ide analogue treatment in a multiethnic cohort of chronic hepatitis B patients: Results after treatment cessation. Clin. Infect. Dis. 65, 680–683. doi: 10.1093/cid/cix353. PMID: 28575292

[B4] Eslam M. Newsome P. N. Sarin S. K. Anstee Q. M. Targher G. Romero-Gomez M. . (2020). A new definition for metabolic dysfunction-associated fatty liver disease: An international expert consensus statement. J. Hepatol. 73, 202–209. doi: 10.1016/j.jhep.2020.03.039. PMID: 32278004

[B5] Fan R. Deng R. Xie Q. Wang F. Liang X. Ma H. . (2025). Novel HBV biomarkers-guided NAs withdrawal strategy promotes HBsAg clearance in Asian CHB patients: A randomized controlled trial. Clin. Gastroenterol. Hepatol., S1542-3565(25)00799–2. doi: 10.1016/j.cgh.2025.09.009. PMID: 40953786

[B6] Gao N. Guan G. Xu G. Wu H. Xie C. Mo Z. . (2023). Integrated HBV DNA and cccDNA maintain transcriptional activity in intrahepatic HBsAg-positive patients with functional cure following PEG-IFN-based therapy. Aliment. Pharmacol. Ther. 58, 1086–1098. doi: 10.1111/apt.17670. PMID: 37644711

[B7] Hatipoglu I. Ercan D. Acilan C. Basalp A. Durali D. Baykal A. T. (2014). Hepatitis B virus e antigen (HBeAg) may have a negative effect on dendritic cell generation. Immunobiology 219, 944–949. doi: 10.1016/j.imbio.2014.07.020. PMID: 25150150

[B8] Hu P. Shang J. Zhang W. Gong G. Li Y. Chen X. . (2018). HBsAg loss with peg-interferon alfa-2a in hepatitis B patients with partial response to nucleos(t)ide analog: New switch study. J. Clin. Transl. Hepatol. 6, 25–34. doi: 10.14218/JCTH.2017.00072. PMID: 29577029 PMC5862996

[B9] Huang D. Wu D. Wang P. Wang Y. Yuan W. Hu D. . (2022). End-of-treatment HBcrAg and HBsAb levels identify durable functional cure after Peg-IFN-based therapy in patients with CHB. J. Hepatol. 77, 42–54. doi: 10.1016/j.jhep.2022.01.021. PMID: 35149125

[B10] Jeng W.-J. Chen Y.-C. Chien R.-N. Sheen I.-S. Liaw Y.-F. (2018). Incidence and predictors of hepatitis B surface antigen seroclearance after cessation of nucleos(t)ide analogue therapy in hepatitis B e antigen-negative chronic hepatitis B. Hepatology 68, 425–434. doi: 10.1002/hep.29640. PMID: 29108132

[B11] Lai C.-L. Wong D. Ip P. Kopaniszen M. Seto W.-K. Fung J. . (2017). Reduction of covalently closed circular DNA with long-term nucleos(t)ide analogue treatment in chronic hepatitis B. J. Hepatol. 66, 275–281. doi: 10.1016/j.jhep.2016.08.022. PMID: 27639844

[B12] Lei Z. Wang L. Gao H. Guo S. Kang X. Yuan J. . (2024). Mechanisms underlying the compromised clinical efficacy of interferon in clearing HBV. Virol. J. 21, 314. doi: 10.1186/s12985-024-02589-3. PMID: 39633459 PMC11619119

[B13] Li M.-H. Yi W. Zhang L. Lu Y. Lu H.-H. Shen G. . (2019). Predictors of sustained functional cure in hepatitis B envelope antigen-negative patients achieving hepatitis B surface antigen seroclearance with interferon-alpha-based therapy. J. Viral Hepat 26 Suppl 1, 32–41. doi: 10.1111/jvh.13151. PMID: 31380582

[B14] Li Y. Yang S. Li C. Ma Z. Zhang M. Zou W. . (2024). Efficacy of short-term Peg-IFN α-2b treatment in chronic hepatitis B patients with ultra-low HBsAg levels: a retrospective cohort study. Virol. J. 21, 231. doi: 10.1186/s12985-024-02512-w. PMID: 39334422 PMC11428405

[B15] Liu J. Li T. Zhang L. Xu A. (2019). The role of hepatitis B surface antigen in nucleos(t)ide analogues cessation among Asian patients with chronic hepatitis B: A systematic review. Hepatology 70, 1045–1055. doi: 10.1002/hep.30474. PMID: 30561829

[B16] Lok A. S. Zoulim F. Dusheiko G. Chan H. L. Y. Buti M. Ghany M. G. . (2020). Durability of hepatitis B surface antigen loss with nucleotide analogue and peginterferon therapy in patients with chronic hepatitis B. Hepatol. Commun. 4, 8–20. doi: 10.1002/hep4.1436. PMID: 31909352 PMC6939500

[B17] Micco L. Peppa D. Loggi E. Schurich A. Jefferson L. Cursaro C. . (2013). Differential boosting of innate and adaptive antiviral responses during pegylated-interferon-alpha therapy of chronic hepatitis B. J. Hepatol. 58, 225–233. doi: 10.1016/j.jhep.2012.09.029. PMID: 23046671

[B18] Ning Q. Han M. Sun Y. Jiang J. Tan D. Hou J. . (2014). Switching from entecavir to PegIFN alfa-2a in patients with HBeAg-positive chronic hepatitis B: a randomised open-label trial (OSST trial). J. Hepatol. 61, 777–784. doi: 10.1016/j.jhep.2014.05.044. PMID: 24915612

[B19] Tang L. S. Y. Covert E. Wilson E. Kottilil S. (2018). Chronic hepatitis B infection: A review. JAMA 319, 1802–1813. doi: 10.1001/jama.2018.3795. PMID: 29715359

[B20] van Campenhout M. J. H. Janssen H. L. A. (2015). How to achieve immune control in chronic hepatitis B? Hepatol. Int. 9, 9–16. doi: 10.1007/s12072-014-9571-3. PMID: 25788374

[B21] Vittal A. Sharma D. Hu A. Majeed N. A. Terry N. Auh S. . (2022). Systematic review with meta-analysis: the impact of functional cure on clinical outcomes in patients with chronic hepatitis B. Aliment. Pharmacol. Ther. 55, 8–25. doi: 10.1111/apt.16659. PMID: 34850415

[B22] Wang T. Tang F. Li F. Chen J. Yan F. Du Q. . (2025). Discussion on the duration of response following HBsAg clearance in patients with chronic hepatitis B treated with PegIFNα-2b. Front. Immunol. 16, 1518048. doi: 10.3389/fimmu.2025.1518048. PMID: 40264777 PMC12011802

[B23] Wen C. Wang Y. Tian H. Lei Y. Wang Z. Cai D. . (2023). Clinical cure induced by pegylated interferon α-2b in the advantaged population of chronic hepatitis B virus infection: a retrospective cohort study. Front. Cell. Infect. Microbiol. 13, 1332232. doi: 10.3389/fcimb.2023.1332232. PMID: 38292859 PMC10824921

[B24] Wu Y. Liu Y. Lu J. Cao Z. Jin Y. Ma L. . (2020b). Durability of interferon-induced hepatitis B surface antigen seroclearance. Clin. Gastroenterol. Hepatol. 18, 514–516.e2. doi: 10.1016/j.cgh.2019.04.020. PMID: 30981007

[B25] Wu F.-P. Yang Y. Li M. Liu Y.-X. Li Y.-P. Wang W.-J. . (2020a). Add-on pegylated interferon augments hepatitis B surface antigen clearance vs continuous nucleos(t)ide analog monotherapy in Chinese patients with chronic hepatitis B and hepatitis B surface antigen ≤ 1500 IU/mL: An observational study. World J. Gastroenterol. 26, 1525–1539. doi: 10.3748/wjg.v26.i13.1525. PMID: 32308352 PMC7152523

[B26] Ye B. Liu X. Li X. Kong H. Tian L. Chen Y. (2015). T-cell exhaustion in chronic hepatitis B infection: current knowledge and clinical significance. Cell Death Dis. 6, e1694. doi: 10.1038/cddis.2015.42. PMID: 25789969 PMC4385920

[B27] Yip T. C.-F. Lok A. S.-F. (2020). How do we determine whether a functional cure for HBV infection has been achieved? Clin. Gastroenterol. Hepatol. 18, 548–550. doi: 10.1016/j.cgh.2019.08.033. PMID: 31446185

[B28] Yip T. C.-F. Wong G. L.-H. Chan H. L.-Y. Tse Y.-K. Lam K. L.-Y. Lui G. C.-Y. . (2019). HBsAg seroclearance further reduces hepatocellular carcinoma risk after complete viral suppression with nucleos(t)ide analogues. J. Hepatol. 70, 361–370. doi: 10.1016/j.jhep.2018.10.014. PMID: 30367899

[B29] Yip T. C.-F. Wong G. L.-H. Wong V. W.-S. Tse Y.-K. Lui G. C.-Y. Lam K. L.-Y. . (2017). Durability of hepatitis B surface antigen seroclearance in untreated and nucleos(t)ide analogue-treated patients. J. Hepatol., S0168-8278(17)32332–2. doi: 10.1016/j.jhep.2017.09.018. PMID: 28989093

[B30] Yu S. Chen J. Wu M. Chen H. Kato N. Yuan Z. (2010). Hepatitis B virus polymerase inhibits RIG-I- and Toll-like receptor 3-mediated beta interferon induction in human hepatocytes through interference with interferon regulatory factor 3 activation and dampening of the interaction between TBK1/IKKepsilon and DDX3. J. Gen. Virol. 91, 2080–2090. doi: 10.1099/vir.0.020552-0. PMID: 20375222

[B31] Yuen M.-F. Lim S.-G. Plesniak R. Tsuji K. Janssen H. L. A. Pojoga C. . (2022). Efficacy and safety of bepirovirsen in chronic hepatitis B infection. N. Engl. J. Med. 387, 1957–1968. doi: 10.1056/NEJMoa2210027. PMID: 36346079

