# Supplementary material for: Sustained HBsAg clearance induced by pegylated interferon α-2b in HBeAg-negative patients with low baseline HBsAg
Source: Front Cell Infect Microbiol. 2026 May 20;16:1803818. doi: 10.3389/fcimb.2026.1803818 (PMC13231276; doi:10.3389/fcimb.2026.1803818)
Supplement: Supplementary file 1 [file Table1.docx]

Table S1. Baseline characteristics of patients receiving different treatment regimens:

|  | All | IFN | NA+IFN | P value |
| --- | --- | --- | --- | --- |
|  | 203 | 99(48.770%) | 104(51.230%) |  |
| Age, years (Mean±SD) | 42(36,50) | 40(35,48) | 44(36,52) | 0.051 |
| Sex, male (n, %) | 136(67.00%) | 61(61.616%) | 75(80.769%) | 0.112 |
| HBsAg reversion (n, %) | 44(21.700%) | 24(24.242%) | 20(19.231%) | 0.312 |
| HBsAg |  |  |  | 0.177 |
| <100 (n, %) | 180(88.670%) | 84(84.848%) | 96(92.307%) |  |
| 100-200 (n, %) | 23(11.330%) | 15(15.152%) | 8(7.693%) |  |
| HBeAg negative (n, %) | 203(100.000%） | 99(100.000%) | 104(100.000%) | 1.000 |
| HBV DNA negative (n, %) | 203(100.000%) | 99(100.000%) | 104(100.000%) | 1.000 |
| ALB, g/L, Median(Q1-Q3) | 47.100(44.900,49.000) | 46.200(44.400,49.000) | 47.100(45.100,49.075) | 0.735 |
| ALT, U/L, Median(Q1-Q3) | 23.000(16.800,32.000) | 19.400(14.000,30.000) | 25.000(18.000,33.000) | **0.004** |
| AST, U/L, Median(Q1-Q3) | 23.000(19.000,27.000) | 21.000(18.000,25.000) | 25.000(21.000,29.000) | **<0.001** |
| ALP, U/L, Median(Q1-Q3) | 66.000(56.000,75.500) | 66.000(56.000,76.000) | 67.000(55.750,75.250) | 0.880 |
| GGT ,U/L, Median(Q1-Q3) | 19.000(15.000,28.000) | 18.000(14.300,28.200) | 19.850(15.000,27.875) | 0.544 |
| TB, μmol/L, Median (Q1-Q3) | 12.100(9.100,15.960) | 12.060(8.300,16.200) | 12.100(9.280,15.415) | 0.786 |
| RBC,10^9^/L, Median(Q1-Q3) | 4.980(4.580,5.340) | 4.820(4.510,5.300) | 5.045(4.680,5.373) | 0.102 |
| WBC,10^9^/L, Median(Q1-Q3) | 5.640(4.780,6.800) | 5.640(4.780,6.570) | 5.625(4.790,6.883) | 0.657 |
| Plt ,10^9^/L, Median(Q1-Q3) | 202(170,236) | 214(181,246) | 198(167,223) | 0.035 |
| AFP, μg/L, Median(Q1-Q3) | 2.510(1.900,3.400) | 2.540(1.840,3.270) | 2.505(1.985,3.475) | 0.427 |
| MASLD (n, %) | 41(20.200%) | 18(18.181%) | 23(22.115%) | 0.462 |
| Fibroscan, Kpa, Median(Q1-Q3) | 5.600(4.500,6.400) | 5.700(4.500,6.400) | 5.400(4.600,6.475) | 0.615 |
| HBcAb, IU/ml, Median(Q1-Q3) | 6.990(6.270,7.820) | 7.000(6.360,7.750) | 6.970(6.140,7.900) | 0.677 |
| HBsAb, IU/ml, Median(Q1-Q3) |  |  |  | 0.779 |
| <100 (n, %) | 113(19.700%) | 54(54.545%) | 59(56.731%) |  |
| ≥1000 (n, %) | 90(35.500%) | 45(45.455%) | 45(43.689%) |  |
| Consolidation therapy |  |  |  | 0.263 |
| <12 weeks (n, %) | 52(13.800%) | 29(29.292%) | 23(22.115%) |  |
| ≥24 weeks (n, %) | 151(32.00%) | 70(70.708%) | 81(77.885%) |  |
| Therapy duration, wk, Median(Q1-Q3) | 36(24,48) | 32(24,40) | 36(24,48) | **0.009** |
| Therapy time before HBsAg loss, wk, Median(Q1-Q3) | 16(12,24) | 12(12,24) | 16(12,24) | 0.125 |
| Follow-up duration, wk, Median(Q1-Q3) | 96(72-120) | 96(72,116) | 96(69,132) | 0.267 |

Abbreviations and instructions: HBcAb, HBcAb levels at end of Peg-IFNα-2b treatment; HBsAb, HBsAb levels at end of Peg-IFNα-2b treatment; Consolidation treatment, continuing therapy by pegylated interferonα-2b after HBsAg loss. SD, standard deviation; TDF, tenofovir disoproxil fumarate; ETV, entecavir; TAF, tenofovir alafenamide; HBeAg, hepatitis B e-antigen; HBsAg, hepatitis B surface antigen; HBV DNA, hepatitis B virus-deoxyribonucleic acid; ALT, alanine aminotransferase; AST, aspartate aminotransferase; ALP, alkaline phosphatase; GGT, gamma-glutamyl tanspepetidase; RBC, red blood cell; WBC, white blood cell; TB, total bilirubin; A, albumin; PLT, platelets; AFP, alpha-fetoprotein; Peg-IFN, pegylated interferon α-2b; NA, nucleoside analog; HBsAb, hepatitis B surface antigen; HBcAb, hepatitis B core antigen; IQR, interquartile range; EOT, end of Peg-IFNα-2b treatment. MAFLD, Metabolic associated fatty liver disease. The values marked in bold signify statistical significance (p<0.05).
