# Supplementary material for: Sustained HBsAg clearance induced by pegylated interferon α-2b in HBeAg-negative patients with low baseline HBsAg
Source: Front Cell Infect Microbiol. 2026 May 20;16:1803818. doi: 10.3389/fcimb.2026.1803818 (PMC13231276; doi:10.3389/fcimb.2026.1803818)
Supplement: Supplementary file 2 [file Table2.docx]

Table S2:

Time Distribution of HBsAg Reversion

| Time (weeks) | HBsAg reversion (n) | Percentage (%) | Cumulative Reversions (n) | Cumulative percentage (%) |
| --- | --- | --- | --- | --- |
| <12 | 1 | 2.72 | 1 | 2.72 |
| 12-24 | 9 | 20.46 | 10 | 23.18 |
| 24-48 | 14 | 31.82 | 24 | 55 |
| 48-72 | 11 | 25 | 35 | 80 |
| 72-96 | 5 | 11.36 | 40 | 91.36 |
| 96-120 | 3 | 5.92 | 43 | 97.28 |
| ≥120 | 1 | 2.72 | 44 | 100 |
