# Supplementary material for: Sustained HBsAg clearance induced by pegylated interferon α-2b in HBeAg-negative patients with low baseline HBsAg
Source: Front Cell Infect Microbiol. 2026 May 20;16:1803818. doi: 10.3389/fcimb.2026.1803818 (PMC13231276; doi:10.3389/fcimb.2026.1803818)
Supplement: Supplementary file 3 [file Table3.docx]

Table S1:

Rate of HBsAg loss following HBsAg clearance

| Time (weeks) | HBsAg reversion (n) | HBsAg loss(n) | Total (n) | HBsAg loss rate (%) |
| --- | --- | --- | --- | --- |
| Total | 44 | 159 | 203 | 78.33 |
| ≥12 | 38 | 159 | 197 | 80.71 |
| ≥24 | 22 | 161 | 183 | 87.98 |
| ≥48 | 16 | 144 | 160 | 90 |
| ≥72 | 6 | 124 | 130 | 95.38 |
| ≥96 | 2 | 100 | 102 | 98.04 |
| ≥120 | 1 | 57 | 58 | 98.28 |
| ≥144 | 0 | 43 | 43 | 100 |
| ≥168 | 0 | 23 | 23 | 100 |
| ≥192 | 0 | 15 | 15 | 100 |
