# Supplementary material for: Sustained HBsAg clearance induced by pegylated interferon α-2b in HBeAg-negative patients with low baseline HBsAg
Source: Front Cell Infect Microbiol. 2026 May 20;16:1803818. doi: 10.3389/fcimb.2026.1803818 (PMC13231276; doi:10.3389/fcimb.2026.1803818)
Supplement: Supplementary file 4 [file Table4.docx]

Table S4:

Characteristics of HBsAg reversion patients

| NO | Sex | Age | HBsAg^1^ | Treatment | HBV DNA^1^ | HBsAg^2^ | HBV DNA^2^ | HBsAb | ALT | AST | Treatment after replase |
| --- | --- | --- | --- | --- | --- | --- | --- | --- | --- | --- | --- |
| 1 | 1 | 38 | 52.91 | TDF+IFN | 0 | 0.3 | 0 | 5.78 | 65 | 36 | TDF |
| 2 | 2 | 40 | 18.89 | ETV+IFN | 0 | 0.06 | 0 | 6.51 | 13 | 26 | ETV |
| 3 | 1 | 38 | 67.33 | TDF+IFN | 0 | 0.26 | 0 | 1.26 | 24 | 27 | TDF |
| 4 | 1 | 47 | 9.95 | TAF+IFN | 0 | 0.07 | 0 | 2.54 | 29 | 19 | TAF |
| 5 | 1 | 31 | 78.99 | IFN | 0 | 0.23 | 0 | 8.32 | 23 | 30 | IFN |
| 6 | 2 | 33 | 148.53 | IFN | 0 | 0.74 | 0 | 5.91 | 15 | 19 |  |
| 7 | 2 | 41 | 85.24 | TAF+IFN | 0 | 0.43 | 0 | 9.23 | 33 | 28 | TAF+IFN |
| 8 | 2 | 35 | 97.37 | TDF+IFN | 0 | 1.81 | 0 | 1.61 | 28 | 24 | TDF+IFN |
| 9 | 1 | 52 | 14.69 | TAF+IFN | 0 | 0.56 | 25.2 | 1.60 | 20 | 22 | IFN |
| 10 | 1 | 44 | 79.55 | IFN | 0 | 1.06 | 0 | .47 | 23 | 19 |  |
| 11 | 2 | 39 | 5.6 | IFN | 0 | 1.25 | 0 | 3.31 | 31 | 42 |  |
| 12 | 1 | 54 | 10.1 | IFN | 0 | 0.14 | 0 | 29.89 | 23 | 19 |  |
| 13 | 1 | 40 | 166.06 | IFN | 0 | 4.64 | 44.9 | .52 | 36 | 24 | ETV |
| 14 | 2 | 55 | 84.6 | ETV+IFN | 0 | 5.54 | 0 | .00 | 11 | 36 | ETV |
| 15 | 1 | 49 | 91.62 | TAF+IFN | 0 | 0.22 | 0 | 18.29 | 54 | 44 | TAF+IFN |
| 16 | 2 | 38 | 60.91 | TAF+IFN | 0 | 0.64 | 0 | 1.80 | 17 | 18 | TAF |
| 17 | 1 | 42 | 3.01 | IFN | 0 | 0.23 | 0 | .34 | 20 | 18 |  |
| 18 | 1 | 37 | 87.5 | TDF+IFN | 0 | 1.24 | 0 | 6.23 | 24 | 23 |  |
| 19 | 1 | 40 | 137.67 | TAF+IFN | 0 | 0.18 | 0 | 5.35 | 29 | 22 | TAF+IFN |
| 20 | 1 | 40 | 100.92 | IFN | 0 | 4.63 | 0 | 2.30 | 15 | 17 |  |
| 21 | 1 | 34 | 170.69 | IFN | 0 | 0.12 | 0 | 2.17 | 29 | 24 | IFN |
| 22 | 1 | 43 | 73.91 | ETV+IFN | 0 | 0.08 | 0 | 9.20 | 15 | 21 | ETV |
| 23 | 1 | 28 | 98.5 | IFN | 0 | 2.79 | 0 | 57.12 | 10 | 18 |  |
| 24 | 1 | 33 | 59.5 | TDF+IFN | 0 | 0.54 | 0 | 2.33 | 23 | 26 | TDF |
| 25 | 1 | 51 | 60.97 | IFN | 0 | 0.17 | 0 | .79 | 31 | 28 |  |
| 26 | 2 | 52 | 11.14 | IFN | 0 | 0.86 | 28.1 | 2.25 | 16 | 20 | ETV |
| 27 | 1 | 40 | 19.62 | IFN | 0 | 0.6 | 0 | 4.32 | 22 | 17 |  |
| 28 | 1 | 47 | 68.48 | IFN | 0 | 1.62 | 23.1 | 5.47 | 25 | 21 | TAF |
| 29 | 1 | 42 | 3.11 | TAF+IFN | 0 | 0.06 | 0 | 1.28 | 29 | 19 | TAF+IFN |
| 30 | 2 | 46 | 162.68 | IFN | 0 | 5.95 | 233.11 | 3.27 | 26 | 29 | TAF |
| 31 | 1 | 52 | 182.64 | ETV+IFN | 0 | 0.62 | 0 | .39 | 28 | 29 | ETV |
| 32 | 1 | 54 | 32.89 | ETV+IFN | 0 | 0.18 | 0 | 14.30 | 27 | 27 | ETV+IFN |
| 33 | 1 | 33 | 122.2 | ETV+IFN | 0 | 0.2 | 0 | 5.50 | 21 | 26 | ETV |
| 34 | 1 | 32 | 9.12 | IFN | 0 | 0.1 | 0 | 2.03 | 11 | 15 |  |
| 35 | 1 | 38 | 8.26 | IFN | 0 | 1.2 | 33.3 | 1.85 | 19 | 23 |  |
| 36 | 2 | 30 | 1.27 | IFN | 0 | 0.32 | 0 | 8.13 | 21 | 24 |  |
| 37 | 1 | 40 | 1.03 | IFN | 0 | 0.15 | 0 | 2.98 | 30 | 23 |  |
| 38 | 2 | 33 | 1 | IFN | 0 | 0.08 | 0 | 1.52 | 10 | 15 |  |
| 39 | 1 | 28 | 151.2 | IFN | 0 | 0.45 | 0 | 3.98 | 12 | 16 |  |
| 40 | 1 | 29 | 113.49 | IFN | 0 | 0.42 | 0 | 30.14 | 15 | 16 | IFN |
| 41 | 1 | 32 | 4.61 | ETV+IFN | 0 | 2.47 | 0 | 6.12 | 21 | 37 | ETV |
| 42 | 1 | 33 | 23.11 | IFN | 0 | 0.17 | 0 | 4.24 | 29 | 13 |  |
| 43 | 1 | 32 | 4.61 | ETV+IFN | 0 | 0.58 | 0 | 5.17 | 12 | 28 | ETV |
| 44 | 2 | 48 | 11.14 | IFN | 0 | 0.28 | 0 | 5.48 | 16 | 19 |  |

Abbreviations: Sex: “1” represents male, “2” represents female. HBsAg^1^, baseline HBsAg levels; HBV DNA^1^, baseline HBV DNA levels; HBsAg^2^, HBsAg levels at reversion; HBV DNA^2^, HBV DNA levels at reversion; HBsAb, HBsAb levels at reversion; ALT. ALT levels at reversion; AST, AST levels at reversion; HBV DNA: “0” presents undetectable; ALT, alanine aminotransferase; AST, aspartate aminotransferase; PLT, platelet; Peg-IFN, pegylated interferon α-2b; NA, nucleoside analog; HBsAb, hepatitis B surface antigen; HBsAg, hepatitis B surface antigen; HBV DNA, hepatitis B virus-deoxyribonucleic acid.
